# Inherited Hemophilia—A Multidimensional Chronic Disease That Requires a Multidisciplinary Approach

**DOI:** 10.3390/life15040530

**Published:** 2025-03-24

**Authors:** Cristina Claudia Tarniceriu, Loredana Liliana Hurjui, Daniela Maria Tanase, Anca Haisan, Razvan Tudor Tepordei, Gabriel Statescu, Simona Alice Partene Vicoleanu, Ancuta Lupu, Vasile Valeriu Lupu, Manuela Ursaru, Alin Horatiu Nedelcu

**Affiliations:** 1 Department of Morpho-Functional Science I, Discipline of Anatomy, “Grigore T. Popa” University of Medicine and Pharmacy, 700115 Iasi, Romania; claudia.tarniceriu@umfiasi.ro (C.C.T.); razvan.tepordei@umfiasi.ro (R.T.T.); gabriel.statescu@umfiasi.ro (G.S.); partene.vicoleanu@umfiasi.ro (S.A.P.V.); alin.nedelcu@umfiasi.ro (A.H.N.); 2 Haematology Clinic, “Sf Spiridon” County Clinical Emergency Hospital Iasi, 700111 Iasi, Romania; 3 Department of Morpho-Functional Science II, Discipline of Physiology, “Grigore T. Popa” University of Medicine and Pharmacy, 700115 Iasi, Romania; loredana.hurjui@umfiasi.ro; 4 Department of Internal Medicine, “Grigore T. Popa” University of Medicine and Pharmacy, 700115 Iasi, Romania; 5 Department of Emergency Medicine, “Grigore T. Popa” University of Medicine and Pharmacy, 700115 Iasi, Romania; anca.haisan@umfiasi.ro; 6 Department of Mother and Child, “Grigore T. Popa” University of Medicine and Pharmacy, 700115 Iasi, Romania; ancuta.ignat1@umfiasi.ro; 7 Department of Surgical Sciences I, “Grigore T. Popa” University of Medicine and Pharmacy, 700115 Iasi, Romania; vasile.lupu@umfiasi.ro (V.V.L.); manuela.ursaru@umfiasi.ro (M.U.); 8 Radiology Clinic, Recovery Hospital, 700661 Iasi, Romania

**Keywords:** hemophilia, arthropathies, comorbidities, joint damage, HCV infection

## Abstract

Background: Articular damage is a marker of hereditary hemophilia, especially affecting the large joints of the upper and lower limbs. This retrospective study aimed to emphasize that hereditary coagulopathies, specifically hemophilia A and B, require a multidisciplinary approach due to their complex nature. The primary objectives of the paper are to determine the prevalence of hemophilic arthropathy among individuals with hemophilia in the northeastern region of Romania, identify the most frequently affected joints, and assess whether there is a correlation between the development of hemophilic arthropathy, the type of hemophilia, and the treatment received. The secondary objectives of the work are to identify a series of particularities regarding the occurrence of the comorbidities depending on the type of hemophilia and the treatment and severity of arthropathies. Materials and Methods: We conducted a retrospective study that included 36 adults with hemophilia A and B. The status of the osteoarticular system was evaluated using the modified Hemophilia Joint Health Score (mHJHS). Twelve joints were evaluated using the following parameters: swelling, duration of swelling, muscle atrophy, joint pain, crepitus on motion, flexion loss, and extension loss. Results and Discussions: The most severe damage was found in the joints of the knees, ankles, elbows, and wrists. In the knees, severe damage was noted significantly more frequently in the right knee (50% vs. 33.3%; *p* = 0.001). In the ankles, a higher frequency of mild damage to the left ankle was noted (44.4% vs. 27.8%; *p* = 0.002). The severe form of hemophilia was correlated with severe joint damage (*p* < 0.05). Comorbidities like cardiovascular disease, obesity, viral infection (HCV infection), and gastrointestinal disease were found in the hemophilia population of our study. All patients with HCV infection had severe joint damage, while 38.5% of patients without HCV infection had mild joint damage, and 30.8% had no joint damage (*p* = 0.001). In all patients with HCV virus infection, the treatment was short-term substitution (intermittent prophylaxis), while in 53.8% of patients without HCV virus infection, the treatment consisted of continuous prophylaxis (*p* = 0.001). Conclusions: It is currently essential to determine methods for comprehensive hemophilia care that involves multidisciplinary medical services necessary for the diagnosis, treatment, and management of the condition and its complications and comorbidities.

## 1. Introduction

Inherited hemophilia is a coagulopathy that is produced by the inherited clotting factor deficiency—factor VIII (hemophilia A) and factor IX (hemophilia B) [1]. The normal range of factor level is 50–150% [2], and in hemophilia, the factor level is <1–40% [2]. According to the severity of factor level deficiency, there are three forms of hemophilia: severe (the factor level is <1%), moderate (the factor level is >1–5%), and mild (the factor level is >5–40%) [3,4]. Clinical symptoms of hemophilia include bleeding complications. Patients with severe or moderate hemophilia suffer from spontaneous bleeds, while those with mild hemophilia only bleed after trauma [3,4]. Approximately 90% of bleeding episodes affect the musculoskeletal system [4]. Patients treated on demand with severe hemophilia bleed in their joints up to 50 times a year [5]. Bleeding tendency is correlated with factor activity [6,7,8]. For patients with severe hemophilia A or B—including those with moderate hemophilia presenting a severe phenotype—the World Federation of Hemophilia strongly recommends continuous prophylaxis to effectively prevent bleeding at all times [1,2]. Together with the evolution of the treatment for hemophilia and the introduction of coagulation factor prophylaxis, the life expectancy of these patients has changed, presenting a series of comorbidities also found in the general population [9,10,11]. All of these aspects have changed the approach to treating people with hemophilia, with the need for a multidisciplinary approach being more and more current. Consequently, the detailed knowledge of the disease and its impact on the osteoarticular system as well as the type of comorbidities encountered in the hemophiliac population is a current topic that allows for the improvement of hemophilia management.

The aim of the present work is to highlight the fact that hereditary coagulopathies, in our case, hemophilia types A and B, require a multidisciplinary approach as it is a multidimensional disease.

The primary aims of this paper are to determine the prevalence of multiple hemophilic arthropathies in individuals with hemophilia in northeastern Romania, identify the most commonly affected joints, and assess whether there is a correlation between the development of hemophilic arthropathies and the type of disease or treatment received.

The secondary objectives of the work are to identify a series of particularities regarding the occurrence of the comorbidities depending on the type of hemophilia, treatment, and severity of arthropathies.

## 2. Materials and Methods

### 2.1. Study Population

We carried out a retrospective observational descriptive study in which the patients with a diagnosis of hemophilia were selected for the target group who presented themselves regularly, between January and December 2024, in the Hematology section of the Emergency Clinical Hospital “St. Spiridon”, Iasi. In this study, we included patients with hemophilia A and B. According to the severity of factor level deficiency, patients were classified into three forms of hemophilia: severe (factor level is <1%), moderate (factor level is =1–5%), and mild (factor level is =5–40%)

The study was conducted in accordance with the Declaration of Helsinki and approved by the Institutional Review Board of St. Spiridon Hospital Iasi. Medical data were collected from medical files of patients, and a consent form was signed by the patients.

### 2.2. Measuring Tools

The first objective was to evaluate the impact of hemophilia on osteoarticular status. The status of the osteoarticular system was evaluated using the modified Hemophilia Joint Health Score (mHJHS). Twelve joints were evaluated (shoulder, elbow, wrist, hip, knee, and ankle). The following parameters were evaluated for each joint: swelling (0—absent; 1—mild; 2—moderate; 3—severe), duration of swelling (0 = <6 months; 1 = >6 months), muscle atrophy (0—none; 1—mild; 2—severe), joint pain (0—no pain; 1—only pain on gentle overpressure or palpation; 2—pain through active range), crepitus on motion (0—none; 1—mild; 2—severe), flexion loss (0 = <5°, 1 = 5–10°, 2 = 11–20°, 3 = >20°), and extension loss (0 = <5°, 1 = 5–10°, 2 = 11–20°, 3 = >20°). The total score for each joint was calculated, and then the degree of severity of each joint was established as follows: absent (0–1 points), mild (2–5 points), moderate (6–10 points), and severe (11–16 points).

The second objective was to evaluate comorbidities (arterial hypertension, ischemic cardiac disease, obesity, or gastrointestinal diseases) and the impact of viral infections (hepatitis C virus—HCV) and correlations with the type of hemophilia and joint damage. For this reason, medical data were collected from medical files.

### 2.3. Statistical Analysis

Descriptive and correlative statistical methods were performed—the F Test and Two samples for Variance (*p* < 0.05) were used to determine the correlation between osteoarticular status and hemophilia (type and form), treatment (continuous prophylaxis or intermittent prophylaxis), and HCV infection.

The χ^2^ test is a non-parametric test that compares 2 or more frequency distributions from the same population; it is applied when the expected events are excluded. Sometimes when a frequency in the calculation formula is small, Yates is applied to correct the formula to obtain a higher estimate of the difference. If the obtained *p* value is lower than the tabular one at the 95% significance threshold, the events are not excluded—in this case, the question arises as to whether they are dependent. This statistical method was used to correlate the association of hemophilia with different comorbidities.

The relative risk (RR) is the ratio between the incidence of a disease in exposed individuals and the incidence of the same disease in non-exposed individuals. The higher the RR, the greater the association between the disease and the risk factor: if the RR is equal to or close to 1, the risk of disease occurrence is the same in the presence or absence of the risk factor; if RR < 1, there is a negative association of the disease with the risk factor (this is not a risk factor, but a protective factor). This was used to evaluate the risk of hemophilia and appearance of different comorbidities.

## 3. Results

### 3.1. Demographic and Clinical Characteristics

In this study, a cohort of 36 adults with hemophilia A and B participated, and their demographic and clinical characteristics are presented in Table 1. Two females and thirty-four males were included, and the most of them have the severe form of hemophilia.

**Figure 1:**
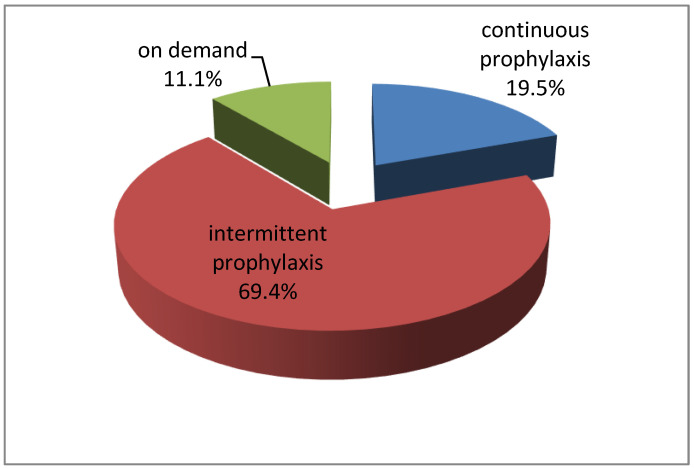
Distribution of hemophilia treatment.

Most patients received short-term substitution treatment (intermittent prophylaxis) (69.4%) (Figure 1).

**Figure 2:**
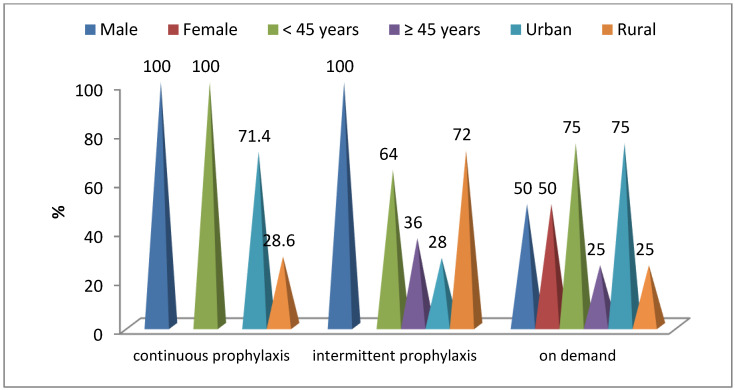
Distribution of patients according to hemophilia treatment.

In patients receiving continuous prophylaxis, the majority were male (100%; *p* = 0.007), under 45 years old (100%; *p* = 0.002), and from urban areas (71.4%; *p* = 0.041). In patients treated with short-term substitution (intermittent prophylaxis), all cases were male (*p* = 0.007), 64% were aged under 45 (*p* = 0.002), and 72% were from rural areas (*p* = 0.041) (Figure 2).

### 3.2. Articular Damage

#### 3.2.1. Distribution of Severity of Articular Damage

**Figure 3:**
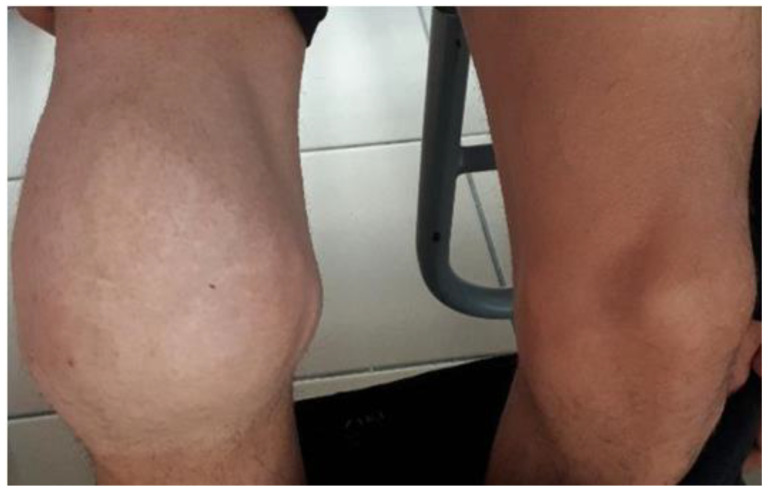
Severe right knee damage in a patient with severe hemophilia A (factor VIII activity < 1%).

Articular damage is the mark of hereditary hemophilia, especially affecting the large joints of the upper and lower limbs. The most severe damage is found in the joints of the knees, ankles, elbows, and wrists (Figure 3).

Knee. In the knees, severe damage was noted significantly more frequently in the right knee (50% vs. 33.3%; *p* = 0.001) (Table 2).

Ankle. In the ankles, a higher frequency of mild damage to the left ankle was noted (44.4% vs. 27.8%; *p* = 0.002) (Table 2).

Hip. Only 25% of patients had damage to the right hip, and 30.6% had damage to the left hip, with the frequency of severe damage being the same in both joints (13.9%; *p* = 0.601) (Table 2).

Wrist. The frequency of severe damage was slightly higher in the right wrist than in the left wrist (11.1% vs. 5.6%; *p* = 0.136) (Table 2).

Elbow. The frequency of severe damage was slightly higher in the right elbow than in the left elbow (19.4% vs. 16.7%; *p* = 0.809) (Table 2).

Shoulder. Only 33.3% of patients suffered damage to the right shoulder, and 22.2% had damage to the left shoulder, and the frequency of severe damage was found only in 8.3% of the right shoulders (*p* = 0.296) (Table 2).

Of the cases with a severe form, 56.5% had at least three affected joints, while moderate and mild forms had only 25% and 20% of cases affecting at least three joints (Table 3).

#### 3.2.2. Correlation Between Form of Hemophilia and Joint Damage

Knee

- A total of 53.6% of patients associated severe hemophilia with severe damage to the right knee, while in patients with mild hemophilia, damage to the right knee was absent in all patients (*p* = 0.028).
- In the left knee, severe hemophilia was associated with severe damage in 39.3% of patients, while in patients with mild hemophilia, damage to the left knee was absent in all patients (*p* = 0.035).

Ankle

- A total of 32.1% of patients associated severe hemophilia with severe damage to the right ankle, while in patients with mild hemophilia, damage to the right ankle was absent in all patients, and in those with moderate hemophilia, damage to the right knee was mild in all patients (*p* = 0.001).
- A total of 25% of patients associated severe hemophilia with severe damage to the left knee, while in patients with mild hemophilia, damage to the left knee was absent in all patients, and in those with moderate hemophilia, damage to the left knee was mild in all patients (*p* = 0.001).

Hip

- A total of 25% of patients associated moderate hemophilia and 14.3% associated severe hemophilia with severe damage to the right hip (*p* = 0.709).
- A total of 25% of patients associated moderate hemophilia and 14.3% associated severe hemophilia with severe damage to the left hip (*p* = 0.605).

Wrist

- A total of 14.3% of patients associated severe hemophilia with severe damage to the right wrist, while mild hemophilia was not associated with damage to the right wrist (*p* = 0.243).
- Severe hemophilia was associated with mild and moderate left wrist damage in 42.8% of patients, while in patients with mild hemophilia, left wrist damage was absent in all patients (*p* = 0.303).

Elbow

- A total of 25% of patients associated severe hemophilia with severe damage to the right elbow, while patients with mild hemophilia did not show damage to the right elbow (*p* = 0.007).
- In the left elbow, severe hemophilia was associated with moderate and severe damage in 53.5% of patients, while in patients with mild hemophilia, damage to the left fist was absent in all patients (*p* = 0.029).

Shoulder

- All patients with mild hemophilia, 75% of patients with moderate hemophilia, and 60.7% of patients with severe hemophilia did not have damage to the right shoulder (*p* = 0.508).
- All patients with mild hemophilia, 75% of patients with moderate hemophilia, and 75% of patients with severe hemophilia did not have damage to the left shoulder (*p* = 0.529).

Our study also examined the relationship between the type of hemophilia, namely A or B, and the degree of joint involvement categorized into mild, moderate, and severe forms. By analyzing these correlations, we aimed to identify potential patterns in disease progression and joint damage severity based on the specific type of hemophilia. The findings from this analysis, including the distribution of joint involvement across different severity levels, are detailed in Table 4.

#### 3.2.3. Correlation Between Treatment and Joint Damage

Continuous prophylaxis refers to the administration of factor VIII or IX during childhood, using standard factor VIII or IX two to three times per week. This regimen was maintained into adulthood, with patients later transitioning to extended half-life factor VIII or IX to improve their quality of life.

Intermittent prophylaxis applies to patients who did not receive continuous factor VIII or IX replacement therapy during childhood, relying instead on on-demand treatment for bleeding episodes. They initiated prophylaxis in adulthood following the national therapeutic protocol.

A notable correlation was observed between the severity of joint damage and prophylactic treatment (Table 5). The most severe joint damage was seen when the patients received only intermittent prophylaxis.

Knee

- A total of 57.1% of patients with continuous prophylaxis did not have right knee damage, while 72% of patients with short-term replacement had severe right knee damage (*p* = 0.001) (Table 5).
- A total of 85.7% of patients with continuous prophylaxis had no left knee damage, while 48% of short-term substitution patients had severe left knee damage (*p* = 0.001) (Table 5).

Ankle

- A total of 42.9% of patients with continuous prophylaxis had mild right ankle involvement, while 36% of short-term substitution patients had severe right ankle involvement (*p* = 0.05) (Table 5).
- A total of 76.7% of patients with continuous prophylaxis and 83.3% of patients with short-term substitution associated mild damage to the left ankle (*p* = 0.019) (Table 5).
- All patients with continuous prophylaxis and 64% of those with short-term substitution did not have a correlation with right hip damage (*p* = 0.148) (Table 5).

Hip

- All patients with continuous prophylaxis and 56% of those with short-term substitution did not associate damage to the left hip (*p* = 0.076) (Table 5).
- All patients with continuous prophylaxis did not present damage to the right hip, while 36% of those with short-term substitution are associated with moderate-severe damage to the right hip (*p* = 0.014) (Table 5).

Wrist

- All patients with continuous prophylaxis did not have damage to the right wrist, while 36% of those with short-term substitution had an association with moderate–severe damage to the right wrist (*p* = 0.014) (Table 5).
- All patients with continuous prophylaxis did not have damage to the left wrist, while 36% of those with short-term substitution had an association with moderate–severe damage to the left wrist (*p* = 0.014) (Table 5).

Elbow

- A total of 28% of patients with short-term substitution associated severe damage to the right elbow, while patients with continuous prophylaxis did not present damage to the right elbow (*p* = 0.025) (Table 5).
- A total of 85.7% of patients with continuous prophylaxis did not have damage to the left elbow, while 60% of the patients with short-term substitution were associated with moderate–severe damage to the left elbow (*p* = 0.002) (Table 5).

Shoulder

- A total of 85.7% of patients with continuous prophylaxis and 56% of patients with short-term substitution did not have an association with right shoulder damage (*p* = 0.147) (Table 5).
- All of the patients with continuous prophylaxis and 68% of those with short-term substitution did not have an association with damage to the left shoulder (*p* = 0.098) (Table 5).

### 3.3. Comorbidities and Hemophilia

The most frequent comorbidity in the hemophilia population in our study was HCV infection (66.7% in hemophilia A and 50% in hemophilia B) (Table 6). The Chi2 test showed that there is a strong association between gastrointestinal disease and hemophilia. RR showed that hemophilia can be a risk factor for cardiovascular disease, viral infection, or gastrointestinal disease (RR > 1) (Table 6).

#### 3.3.1. Correlation Between Form of Hemophilia and HCV Infection

**Figure 4:**
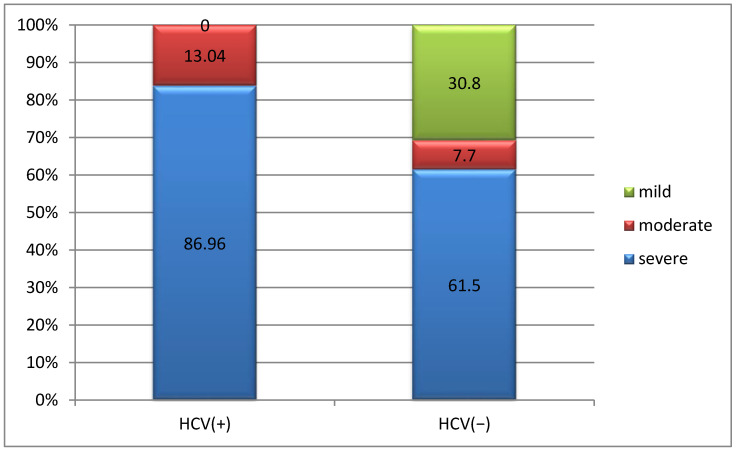
Correlation between forms of hemophilia and HCV infection.

Severe hemophilia was significantly correlated with the presence of HCV infection (86.96%; *p* = 0.011) (Figure 4).

#### 3.3.2. Correlation Between Joint Damage and HCV Infection

**Figure 5:**
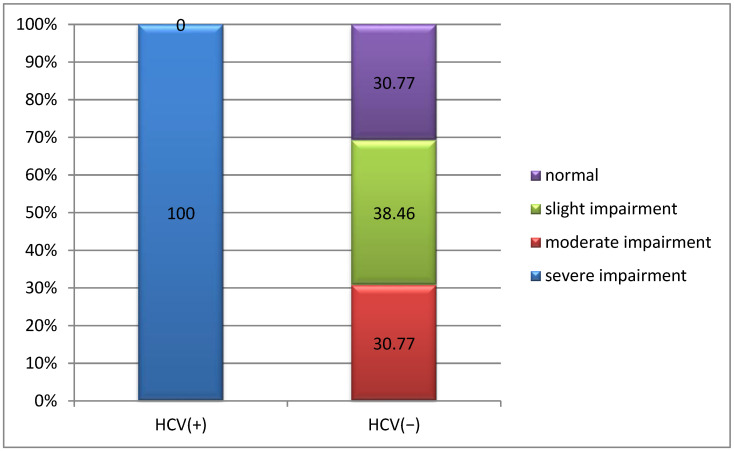
Correlation between severity of joint damage and HCV infection.

All patients with HCV infection had severe joint damage, while 38.46% of patients without HCV infection had mild joint damage and 30.77% had no joint damage (*p* = 0.001) (Figure 5).

#### 3.3.3. Correlation Between Treatment and HCV Infection

**Figure 6:**
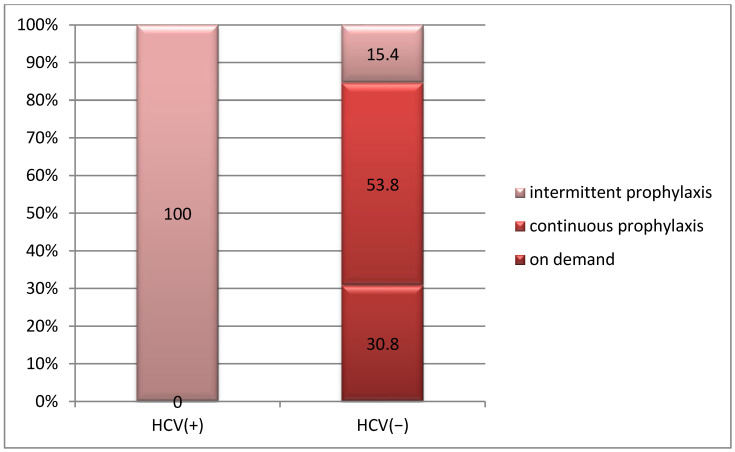
Correlation between hemophilia treatment and HCV infection.

In all patients with HCV virus infection, the treatment was short-term substitution, while in 53.8% of patients without HCV virus infection, the treatment consisted of continuous prophylaxis (*p* = 0.001) (Figure 6).

## 4. Discussion

Our study is the first study to characterize a segment of the hemophiliac population from the northeastern region of Romania, and the goal was to clinically evaluate the osteoarticular status as well as the presence of other comorbidities. In this study, the distribution of cases according to the type of hemophilia was much higher for hemophilia type A, namely 83.3% of cases, while hemophilia B cases had a weight of 16.7%, and 63.9% of cases were the severe form of hemophilia. This uneven distribution is in accordance with the data from the specialized literature where hemophilia A cases comprise 80–85% and hemophilia B cases comprise 15–20% [1,12]. Two women, daughters of a father with hemophilia, experienced recurrent bleeding episodes, including heavy menstruation, and had factor VIII activity below 40%, indicating a mild form of hemophilia A. They were genetically tested and are carriers of the hemophilia gene presenting the inversion of intron 22 on the X chromosome. In the US, for every man diagnosed with hemophilia, there are 1.6 women who carry the hemophilia gene. The US Centers for Disease Control and Prevention notes that among hemophilia cases, women account for 0.5% of severe cases, 1.4% of moderate cases, and about 20% of mild cases [11]. Although originally female carriers were long thought to be asymptomatic, this misconception is now countered by the many cases where a history of bleeding was seen in female carriers, even among those with normal levels of FVIII and IX. So, experts in the field of hemophilia and the International Society of Thrombosis and Hemostasis have introduced new nomenclature in which they classify carrier women into five categories: women/girls with moderate/severe/mild hemophilia, (FVIII/IX > 0.05 and <0.40 IU/mL, 0.01–0.05 IU/mL, and <0.01 IU/mL), symptomatic, and asymptomatic (FVIII/IX > 0.40 IU/mL with or without bleeding phenotype) [12,13]. This new nomenclature is intended to improve diagnosis and management and to provide uniform terminology in clinical research, and in our study we found two cases of the mild form of hemophilia A in a female that required only on-demand treatment.

In our study, a clinical evaluation of the osteoarticular status was performed in patients with hemophilia, with the large joints of the upper and lower limbs being evaluated. mHJHS was also applied to the hip, wrist, and shoulder joints even though they are less involved in the occurrence of hemophilic arthropathy. The joints most frequently involved and with the most severe destruction were the joints of the lower limbs, knees, and ankles, but still, joint damage was found to a lesser degree at the level of the other joints, including the hip and shoulder. Since the knee joint and the ankle joint are loading and force transfer joints, they are more affected than the elbow joint, which can only be affected in the case of overuse of the upper limb or in activities that predominately use the hands and arms. Bleeding is much more common, as we mentioned previously, in large joints such as the knee, ankle, and elbow, which are monoplanar joints in contrast to the hip and shoulder joints, which are multiplanar [14,15].

Patients with the severe form of hemophilia (factor VIII/IX activity < 1% of normal) are more prone to joint damage than patients with mild or moderate hemophilia [16], and our study confirms that.

Most of our patients did not receive prophylaxis with factor VIII/IX during childhood, which negatively impacted their osteoarticular status compared to those who received prophylactic treatment. Research consistently supports that continuous prophylaxis with factor replacement therapy is more effective in reducing the development of arthropathy than episodic treatment, such as on-demand therapy or intermittent prophylaxis [17,18,19].

The results of our study show that patients who received continuous prophylaxis with coagulation factor have a smaller number of affected joints but continue to present joint damage, with the most common joint involved in this category of patients being the ankle. A recent study showed that the most affected joint in a German person with hemophilia that received factor VIII/IX prophylaxis is still the ankle (41%), followed by the knee (27%) and the elbow (11%). The most common source of pain is also the ankle joint (32%) [20]. Bleeding episodes have become rarer with the improvement in therapy and the introduction of prophylaxis, so lately, there has been an increase in the involvement of the ankle joint compared to the knee probably due to the thinner layer of cartilage. In addition, the low expression of tissue factor at the level of normal joints and muscle tissue affects the formation of a clot, contributing to the predisposition of bleeding at this level [21,22]. Hemophilic arthropathy remains a clinical reality for patients with hemophilia, even in the presence of continuous prophylaxis with a coagulation factor, which is why the approach of a personalized prophylaxis according to the patient’s lifestyle and hemorrhagic phenotype represents the current standard of management. Also increasing the quality of life for these patients requires a multidisciplinary approach to hemophilic arthropathy, which includes the orthopedic surgeon, the hematologist, the physiotherapist, and the kinesiotherapist.

As a result of the improvement in coagulation factor replacement therapy, the life expectancy of these patients increased, which led to the simultaneous appearance of various comorbidities that are found in the general population (cardiovascular diseases, metabolic, gastrointestinal diseases, and viral infections). Through its results, our study shows the presence of these types of comorbidities in hemophiliac patients. A non-random association was demonstrated between gastrointestinal diseases and hemophilia (chi2 test, *p* < 0.05). A recent publication showed that gastrointestinal diseases can occur in patients with hemophilia A/B and should not be solely attributed to hemophilia. Optimal preventive management, including the treatment of helicobacter pylori infection, the management of comorbidities, the avoidance of drugs that cause bleeding, close monitoring for gastrointestinal symptoms, and a multidisciplinary approach and optimal timing of endoscopy are recommended for effective management in this population [23,24]. The relative risk calculated in our study shows that the association with hemophilia can contribute to increasing the risk of cardiovascular, metabolic, and gastrointestinal diseases (chronic ischemic heart disease, hypertension, obesity, and gastro-duodenal ulcer) (RR > 1). All of this indicates that sedentarism becomes a health problem for these patients as well. A lack of physical activity because of the presence of arthropathies results in weight gain with the appearance of metabolic and cardiovascular diseases. All of this represents a vicious cycle that aggravates hemophilic arthropathy and decreases the quality of life of these patients, which is why the multidisciplinary approach has become the current approach, and these patients should no longer be seen as being protected from the occurrence of cardiovascular diseases because of the presence of hemophilia.

Among the comorbidities encountered in our study, infection with the virus hepatitis C is the most frequently encountered. This is found in patients who received intermittent prophylaxis with coagulation factors and received transfusions with frequent blood products (plasma, cryoprecipitate, and erythrocyte mass). In patients with continuous prophylaxis who received recombinant coagulation factor products in particular, no HCV infections were detected, which demonstrates and confirms the data from the specialized literature that this infection is directly related to the type of treatment administered [25,26,27]. HCV infection was associated with a severe form of hemophilia and with severe joint destruction, and from here, the idea of an arthropathy induced by HCV infection is extrapolated. Hepatitis C-related arthropathy is one of the most common extrahepatic manifestations of HCV infection [28,29,30]. The exact mechanism by which HCV infection triggers arthritis has not been determined, but it is thought to be a local inflammatory response to synovial tissue damage caused directly by viral invasion or indirectly by the deposition of cryoglobulin-induced immune complexes in synovial fluid [28,29,30].

### 4.1. The Clinical Implications of the Study

The results of this study have several important implications for clinical practice and future research. First, the study highlights bleeding in hemophilia carriers, suggesting the need for the diagnosis of hemophilia in female patients. Secondly, it emphasizes the need for continuous prophylaxis and the personalization of treatment regimens for hemophilia patients, considering factors such as the severity of the condition, bleeding risk, and response to therapy, because hemophilic arthropathy still represents a clinical reality. Third, our study shows that comorbidities in hemophilia patients become frequent as in the general population and that knowledge of hematological pathology is required, in addition to a multidisciplinary approach.

### 4.2. Study Limitations

The single-center design and relatively small sample size limit the generalizability of the findings to other populations. Future studies should consider the correlation of the clinical assessment of arthropathies with the ultrasonographic one.

## 5. Conclusions

Patients with hemophilia continue to have symptoms secondary to hemophilic arthropathy and other comorbidities. It has become essential to determine a comprehensive method of hemophilia care that involves multidisciplinary medical services necessary for the diagnosis, treatment, and management of the condition and its complications and comorbidities.

## Figures and Tables

**Table 1:** Demographic and clinical features of patients.

Characteristics	* n *	%
* Gender *		
Male Female	34 2	94.4 5.6
* Age *		
<45 years old ≥45 years old	17 19	47.2 52.8
* Urban vs. rural *		
Urban Rural	15 21	41.7 58.3
* Type of hemophilia *		
Type A Type B	30 6	83.3 16.7
* Forms of hemophilia *		
Mild Moderate Severe	4 4 28	11.1 11.1 77.8
* Treatment *		
On demand Continuous prophylaxis Intermittent prophylaxis	4 7 25	11.1 19.5 69.4

**Table 2:** Distribution of patients according to joint damage.

Joint	Absent	Mild	Moderate	Severe	*p*
Right knee	10 (27.8%)	2 (5.6%)	6 (16.7%)	18 (50.0%)	0.001
Left knee	12 (33.3%)	4 (11.1%)	8 (22.2%)	12 (33.3%)	
Right ankle	7 (19.4%)	10 (27.8%)	10 (27.8%)	9 (25.0%)	0.002
Left ankle	7 (19.4%)	16 (44.4%)	6 (16.7%)	7 (19.4%)	
Right hip	27 (75,0%)	3 (8.3%)	1 (2.8%)	5 (13.9%)	0.601
Left hip	25 (69.4%)	4 (11.1%)	2 (5.6%)	5 (13.9%)	
Right wrist	21 (58.3%)	6 (16.7%)	5 (13.9%)	4 (11.1%)	0.136
Left wrist	21 (58.3%)	6 (16.7%)	7 (19.4%)	2 (5.6%)	
Right elbow	13 (36.1%)	6 (16.7%)	10 (27.8%)	7 (19.4%)	0.809
Left elbow	14 (38.9%)	7 (19.4%)	9 (25.0%)	6 (16.7%)	
Right shoulder	24 (66.7%)	3 (8.3%)	6 (16.7%)	3 (8.3%)	0.296
Left shoulder	28 (77.8%)	3 (8.3%)	5 (13.9%)	-	

**Table 3:** Severity of joints affected and number of joints that have severe damage.

Joint Score	Study		At Least 3 Joints Affected	
	No	%	*n*	%
Absent	4	11.1		
Mild	5	13.9	1	20.0
Moderate	4	11.1	1	25.0
Severe	23	63.9	13	56.5

**Table 4:** Correlation between types of hemophilia and joint damage.

Joint Damage	Hemophilia A			Hemophilia B			*p*
	Mild	Moderate	Severe	Mild	Moderate	Severe	
Right knee	6.7%	16.7%	50.0%	0.0%	16.7%	50.0%	0.848
Left knee	10.0%	26.7%	36.7%	16.4%	0.0%	16.7%	0.050
Right ankle	16.7%	30.0%	30.0%	83.3%	16.7%	0.0%	0.007
Left ankle	36.7%	20.0%	23.3%	83.3%	0.0%	0.0%	0.048
Right hip	10.0%	3.3%	13.3%	0.0%	0.0%	16.7%	0.668
Left hip	10.0%	6.7%	13.3%	16.7%	0.0%	16.7%	0.812
Right wrist	20.0%	13.3%	13.3%	0.0%	16.7%	0.0%	0.223
Left wrist	20.0%	20.0%	6.7%	0.0%	0.0%	16.7%	0.302
Right elbow	16.7%	30.0%	23.3%	16.7%	16.7%	0.0%	0.214
Left elbow	13.3%	30.0%	20.0%	50.0%	0.0%	0.0%	0.040
Right shoulder	10.0%	16.7%	10.0%	0.0%	16.7%	0.0%	0.481
Left shoulder	10.0%	13.3%	0.0%	0.0%	16.7%	0.0%	0.560

**Table 5:** Correlation between types of treatment and joint damage.

Joint Damage	Treatment						*p*
	Continuous Prophylaxis			Intermittent Prophylaxis			
	Mild	Moderate	Severe	Mild	Moderate	Severe	
Right knee	14.3%	28.6%	0.0%	4.0%	16.0%	72.0%	0.001
Left knee	14.3%	0.0%	0.0%	12.0%	32.0%	48.0%	0.001
Right ankle	42.9%	28.6%	0.0%	28.0%	32.0%	36.0%	0.050
Left ankle	36.7%	20.0%	23.3%	83.3%	0.0%	0.0%	0.019
Right hip	0.0%	0.0%	0.0%	12.0%	4.0%	20.0%	0.148
Left hip	0.0%	0.0%	0.0%	16.0%	8.0%	20.0%	0.076
Right wrist	0.0%	0.0%	0.0%	24.0%	20.0%	16.0%	0.014
Left wrist	0.0%	0.0%	0.0%	24.0%	28.0%	8.0%	0.014
Right elbow	14.3%	14.3%	0.0%	20.0%	36.0%	28.0%	0.025
Left elbow	14.3%	0.0%	0.0%	24.0%	36.0%	24.0%	0.002
Right shoulder	14.3%	0.0%	0.0%	8.0%	24.0%	12.0%	0.147
Left shoulder	0.0%	0.0%	0.0%	12.0%	20.0%	0.0%	0.098

**Table 6:** Correlation between hemophilia and comorbidities (_A_—hemophilia A; _B_—hemophilia B).

Comorbidities	Hemophilia A (*n* = 30)		Hemophilia B (*n* = 6)		Chi2 Test *p*	RR	IC95%
	*n*	%	*n*	%			
HCV infection	20	66.7	3	50.0	0.369	1.77 _A_	0.42–7.53
Arterial hypertension	8	26.7	0	0.0	0.193	1.27 _A_	1.05–1.54
Ischemic cardiac disease	5	16.7	0	0.0	0.378	1.24 _A_	1.04–1.47
Obesity	5	16.7	2	33.3	0.329	1.21 _B_	0.74–1.97
Gastrointestinal disease	2	6.7	2	33.3	0.048	7.00 _B_	0.76–6.46
Others	4	13.3	0	0.0	0.465	1.23 _A_	1.04–1.45

## Data Availability

All relevant data are contained within the manuscript. The raw data supporting the conclusions of this article will be made available by the authors upon request.
